# Editorial: PsychOncology: clinical psychology for cancer patients—Cancer: the key role of clinical psychology

**DOI:** 10.3389/fpsyg.2015.00947

**Published:** 2015-07-06

**Authors:** Lorys Castelli, Gianluca Castelnuovo, Riccardo Torta

**Affiliations:** ^1^Department of Psychology, University of TurinTurin, Italy; ^2^Department of Psychology, Catholic University of MilanMilan, Italy; ^3^Psychology Research Laboratory, Istituto Auxologico Italiano Istituto di Ricovero e Cura a Carattere ScientificoOspedale San Giuseppe, Verbania, Italy; ^4^Clinical Psychology and Psycho-Oncology Unit, Department of Neuroscience, University of TurinTurin, Italy

**Keywords:** cancer, psychoncology, psychological distress, depression, caregiver

Psychological issues take on great importance in oncology settings from the communication of the diagnosis to the management of the end-of-life phase (Baider and Surbone, 2014; Chaturvedi et al., 2014; Grassi et al., 2015). When diagnosed with cancer, about 30% of patients can suffer from psychological distress or other important mental health conditions (Singer et al., 2010, 2013; Mitchell et al., 2011; Vehling et al., 2012).

The papers of the present research topic give an insight into what taking care of a cancer patient means at the individual, relational, and sociocultural levels. The topics of the papers range from the importance of personality and the psychological distress/psychopathology which cancer patients may experience to the essential role of the caregiver and the relevance of socio-cultural factors.

It is now well-proven that psychological distress is a key variable in taking care of cancer patients. A meta-analytic review investigating the longitudinal associations between stress and cancer evidenced in 165 studies that stress-related psychosocial factors are associated with higher cancer incidence in initially healthy populations (Chida et al., 2008). A further 330 studies showed that stress in patients with cancer was related to a poorer rate of survival (Chida et al., 2008).

Moreover, individuals with poor social support experienced greater tumor growth and progression, due to increased pro-inflammatory mechanisms (Lutgendorf and Sood, 2011). Poor social support causes an increase of angiogenesis, essential for cancer growth, due to the stress-related production of a cytokine (IL6) acting on the vascular endothelial growth factor.

Depression, too, could increase the risk of relapses following cancer treatment and reduce adherence to antineoplastic therapy (Reich, 2008). Moreover, since pain and mood disorders share several pathogenetic mechanisms (Torta and Ieraci, 2013), depression could result in a reduction of the pain threshold (Torta and Munari, 2010). An inflammatory background, mainly due to proinflammatory cytokines, is a central factor that links depression, distress, pain, and cognitive impairment (“chemo-fog”) in cancer patients (Krishnadas and Cavanagh, 2012).

The first important step in oncology practice should therefore be screening for psychological distress using well-established clinical instruments, such as the Distress Thermometer (DT) and the Hospital Anxiety and Depression Scale (Castelli et al., 2011; Grassi et al., 2015). A recent PET study on mixed cancer patients highlighted that the DT scores correlated with the activity of brain areas typically involved in stress response: the higher the DT score, the higher the brain metabolism in brain areas involved in stress, such as the hypothalamus (Castelli et al., 2013).

As stressed by Grassi et al. (2015), after the first screening step, patients reporting relevant symptoms should be assessed for more in-depth psychosocial issues. Among these issues, personality traits, coping, attachment style and closeness of personal relationships take on particular importance (Saita et al., 2015). The use of more specific psychological and psychosocial instruments allow the clinician not only to quantify psychological distress but also to better characterize the patients from a psychological standpoint and thus provide a more effective treatment.

Civilotti et al. (2015) showed a high percentage of post-traumatic symptomatology (about 60%) in a sample of 92 mixed cancer patients, highlighting how the psychological reaction to cancer may be ascribed to the traumatic spectrum, and can result in dissociative symptoms. The results of the study by Saita et al. (2015) showed that patients who have personality traits of assertiveness and social anxiety tend to use active coping strategies, i.e., Fighting Spirit. Also, the perceived strength of relationships was found to be predictive of using an active coping style (Saita et al., 2015).

The clinical relevance of the patient's close relationships was also stressed by Gritti's contribution (Gritti, 2015). The author elucidates the importance of meeting the patient's family, and clearly lists the themes that should be addressed and the conversational strategies to be adopted.

The key role played by the caregiver should lead cancer care services to actively include caregiver psychological distress at the core of their health policy. As reported by Baider and Surbone (2014), 40–70% of caregivers have been estimated to have clinically relevant symptoms of depression [National Alliance for Caregiving, (NAC) and AARP, 2008, 2009]. To ignore this evidence would negatively impact on not only the caregiver, but also the care receiver. Healthcare systems can no longer ignore the importance of caring for the caregivers in our endeavors to heal cancer. The universality of cancer and its impact on every aspect of life, highlights the central role of clinical psychology in addressing both social and cultural factors. The papers by Chaturvedi et al. (2014); Baider and Surbone (2014), and Grassi et al. (2015) stressed the key role of the social dimension. As stated by Baider and Surbone (2014), “studies have shown that cultural beliefs and social norms play a part in influencing family emotions and behavior with respect to cancer perception and in their appraisals of illness and health.” Family health-culture norms and beliefs about health and illness, should be taken into account in order to provide appropriate and effective treatment and care to patients and their families (Chaturvedi et al., 2014).

Finally, Cormio et al. (2014) investigated posttraumatic growth in caregivers of cancer patients. Once again, the results highlighted that cancer could result in relevant changes not only in the individual, but also in the whole family system. Specifically, they found that both the patient and caregiver may experience posttraumatic growth after the cancer experience. In some cases, patients and caregivers were able to recognize positive elements and growth even in the dramatic experience of a diagnosis of cancer (Cormio et al., 2014).

Psycho-oncology does not have a long history, but in more than 30 years it has “produced a model in which the psychological domain has been integrated, as a subspecialty, into the disease-specific specialty of oncology. As such, the field today contributes to the clinical care of patients and families, to the training of staff in psychological management, and to collaborative research” (Holland and Weiss, 2010, p. 3). It is imperative that research and practice in clinical psychology evolves in parallel with medical developments, in order to ensure an integrated and evidence-based approach within this field (Castelnuovo, 2010).

## Funding

Lorys Castelli was supported by University of Turin grants (“Ricerca scientifica finanziata dall'Università” Linea Generale and Linea Giovani).

### Conflict of interest statement

The authors declare that the research was conducted in the absence of any commercial or financial relationships that could be construed as a potential conflict of interest.

## References

[B1] Baider L. Surbone A. (2014). Universality of aging: family caregivers for elderly cancer patients. Front. Psychol. 5:744. 10.3389/fpsyg.2014.00744 25076927 PMC4097431

[B2] Castelli L. Binaschi L. Caldera P. Mussa A. Torta R. (2011). Fast screening of depression in cancer patients: the effectiveness of the HADS. Eur. J. Cancer Care (Engl). 20, 528–533. 10.1111/j.1365-2354.2010.01217.x 20738391

[B3] Castelli L. Tonello D. D'Agata F. Caroppo P. Baudino B. Zotta M.. (2013). The neurobiological basis of the Distress Thermometer: a PET study in cancer patients. Stress Health. [Epub ahead of print]. 10.1002/smi.2546 24677552

[B4] Castelnuovo G. (2010). No medicine without psychology: the key role of psychological contribution in clinical settings. Front. Psychol. 1:4. 10.3389/fpsyg.2010.00004 21833187 PMC3153736

[B5] Chaturvedi S. K. Strohschein F. J. Saraf G. Loiselle C. G. (2014). Communication in cancer care: psycho-social, interactional, and cultural issues. A general overview and the example of India. Front. Psychol. 5:1332. 10.3389/fpsyg.2014.01332 25452741 PMC4233911

[B6] Chida Y. Hamer M. Wardle J. Steptoe A. (2008). Do stress-related psychosocial factors contribute to cancer incidence and survival? Nat. Clin. Pract. Oncol. 5, 466–475. 10.1038/ncponc1134 18493231

[B7] Civilotti C. Castelli L. Binaschi L. Cussino M. Tesio V. Di Fini G.. (2015). Dissociative symptomatology in cancer patients. Front. Psychol. 6:118. 10.3389/fpsyg.2015.00118 25759675 PMC4338656

[B8] Cormio C. Romito F. Viscanti G. Turaccio M. Lorusso V. Mattioli V. (2014). Psychological well-being and post-traumatic growth in caregivers of cancer patients. Front. Psychol. 5:1342. 10.3389/fpsyg.2014.01342 25477853 PMC4238371

[B9] Grassi L. Caruso R. Sabato S. Massarenti S. Nanni M. G. the UniFe Psychiatry Working Group Coauthors. (2015). Psychosocial screening and assessment in oncology and palliative care settings. Front. Psychol. 5:1485. 10.3389/fpsyg.2014.01485 25709584 PMC4285729

[B10] Gritti P. (2015). The family meetings in oncology: some practical guidelines. Front. Psychol. 5:1552. 10.3389/fpsyg.2014.01552 25653629 PMC4299440

[B11] Holland J. C. Weiss T. R. (2010). History of Psycho-oncology, in Psycho-Oncology, eds Holland J. C. Breitbart William S. Jacobsen Paul B. Lederberg Marguerite S. Loscalzo Matthew J. McCorkle R. (New York, NY: Oxford University Press), 3–12.

[B12] Krishnadas R. Cavanagh J. (2012). Depression: an inflammatory illness? J. Neurol. Neurosurg. Psychiatry 83, 495–502. 10.1136/jnnp-2011-301779 22423117

[B13] Lutgendorf S. K. Sood A. K. (2011). Biobehavioral factors and cancer progression: physiological pathways and mechanisms. Psychosom. Med. 73, 724–730. 10.1097/PSY.0b013e318235be76 22021459 PMC3319047

[B14] Mitchell A. J. Chan M. Bhatti H. Halton M. Grassi L. Johansen C.. (2011). Prevalence of depression, anxiety, and adjustment disorder in oncological, haematological, and palliative care settings: a meta-analysis of 94 interview-based studies. Lancet Oncol. 12, 160–174. 10.1016/S1470-2045(11)70002-X 21251875

[B15] National Alliance for Caregiving (NAC) and AARP (2008, 2009). Caregiving in the U.S. Washington, DC.

[B16] Reich M. (2008). Depression and cancer: recent data on clinical issues, research challenges and treatment aproaches. Support. Care Cancer 20, 353–359. 10.1097/cco.0b013e3282fc734b 18525327

[B17] Saita E. Acquati C. Kayser K. (2015). Coping with early stage breast cancer: examining the influence of personality traits and interpersonal closeness. Front. Psychol. 6:88. 10.3389/fpsyg.2015.00088 25699003 PMC4318273

[B18] Singer S. Das-Munshi J. Brähler E. (2010). Prevalence of mental health conditions in cancer patients in acute care—a meta-analysis. Ann. Oncol. 21, 925–930. 10.1093/annonc/mdp515 19887467

[B19] Singer S. Ehrensperger C. Briest S. Brown A. Dietz A. Einenkel J.. (2013). Comorbid mental health conditions in cancer patients at working age—prevalence, risk profiles, and care uptake. Psychooncology 22, 2291–2297. 10.1002/pon.3282 23494948

[B20] Torta R. G. V. Ieraci V. (2013). Depressive disorders and pain: a joint model of diagnosis and treatment. J. Pain Relief S2:003 10.4172/2167-0846.S2-003

[B21] Torta R. G. V. Munari J. (2010). Symptom cluster: depression and pain. Surg. Oncol. 19, 155–159. 10.1016/j.suronc.2009.11.007 20106657

[B22] Vehling S. Koch U. Ladehoff N. Schön G. Wegscheider K. Heckl U.. (2012). Prävalenz affektiver und angststörungen bei krebs: systematischer literature review und metaanalyse. Psychother. Psychosom. Med. Psychol. 62, 249–258. 10.1055/s-0032-1309032 22585582

